# Sequencing of a Chinese tetralogy of Fallot cohort reveals clustering mutations in myogenic heart progenitors

**DOI:** 10.1172/jci.insight.152198

**Published:** 2022-01-25

**Authors:** Clara Sze Man Tang, Mimmi Mononen, Wai-Yee Lam, Sheng Chih Jin, Xuehan Zhuang, Maria-Mercè Garcia-Barcelo, Qiongfen Lin, Yujia Yang, Makoto Sahara, Elif Eroglu, Kenneth R. Chien, Haifa Hong, Paul Kwong Hang Tam, Peter J. Gruber

**Affiliations:** 1 Department of Surgery, Li Ka Shing Faculty of Medicine, The University of Hong Kong, Hong Kong SAR, China.; 2 Dr. Li Dak-Sum Research Centre, The University of Hong Kong-Karolinska Institutet Collaboration in Regenerative Medicine, Hong Kong SAR, China.; 3 Department of Cell and Molecular Biology, Karolinska Institutet, Stockholm, Sweden.; 4 Department of Genetics, Washington University School of Medicine, St. Louis, Missouri, USA.; 5 Department of Surgery, Yale University School of Medicine, New Haven, Connecticut, USA.; 6 Department of Cardiothoracic Surgery, Shanghai Children’s Medical Center, Shanghai Jiao Tong University School of Medicine, Shanghai, China.

**Keywords:** Cardiology, Development, Cardiovascular disease, Genetic variation

## Abstract

Tetralogy of Fallot (TOF) is the most common cyanotic heart defect, yet the underlying genetic mechanisms remain poorly understood. Here, we performed whole-genome sequencing analysis on 146 nonsyndromic TOF parent-offspring trios of Chinese ethnicity. Comparison of de novo variants and recessive genotypes of this data set with data from a European cohort identified both overlapping and potentially novel gene loci and revealed differential functional enrichment between cohorts. To assess the impact of these mutations on early cardiac development, we integrated single-cell and spatial transcriptomics of early human heart development with our genetic findings. We discovered that the candidate gene expression was enriched in the myogenic progenitors of the cardiac outflow tract. Moreover, subsets of the candidate genes were found in specific gene coexpression modules along the cardiomyocyte differentiation trajectory. These integrative functional analyses help dissect the pathogenesis of TOF, revealing cellular hotspots in early heart development resulting in cardiac malformations.

## Graphical abstract

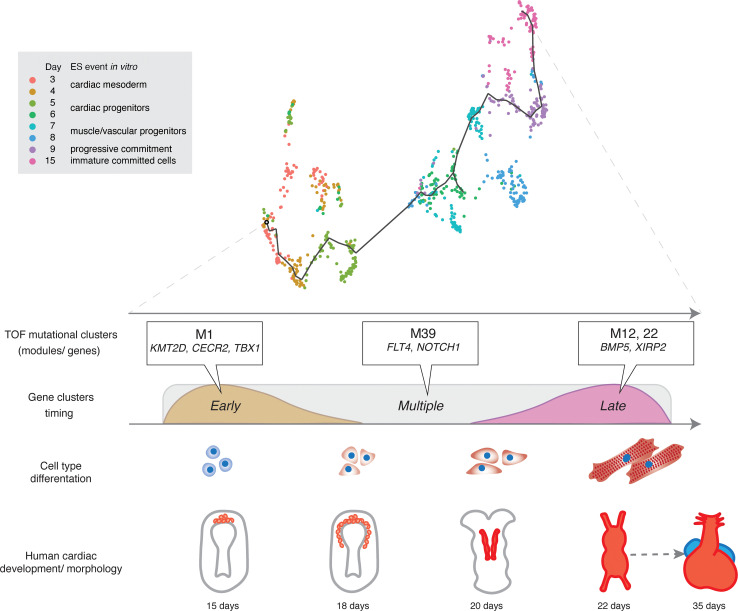

## Introduction

Congenital heart defects (CHDs) have an incidence of approximately 1% at birth and remain the leading cause of infant mortality worldwide (1). Tetralogy of Fallot (TOF) is the most common form of cyanotic CHD, accounting for 10% of all CHDs, and is characterized by 4 anatomic features: (i) a ventricular septal defect, (ii) pulmonary artery stenosis, (iii) an overriding aorta, and (iv) right ventricular hypertrophy (2).

Developmentally, malalignment of the outflow tract is the primary anatomic defect in TOF, from which other features follow (2). Morphogenesis of the outflow tract is dependent upon early progenitor populations of second heart field and neural crest, though the relative contributions of each are incompletely understood (3). Obstruction of the right ventricular outflow tract restricts pulmonary blood flow, resulting in right-to-left shunting and cyanosis. TOF is occasionally seen in combination with other significant cardiac defects, such as pulmonary atresia, absent pulmonary valve, and atrioventricular septal defect, all of which carry considerably higher clinical risk than isolated TOF. Despite the improving surgical outcomes, patients remain at substantial risk for cardiac arrhythmias, pulmonary regurgitation, right ventricular failure, and neurodevelopmental deficits with increasing age. Variations in postoperative responses are unpredictable. Even in patients with similar anatomic features and nearly identical surgical procedures, the ability to predict postoperative course is limited, emphasizing the need for a more complete understanding of the pathogenesis of TOF (4).

Prevailing evidence suggests a strong role for genetic predisposition in the pathogenesis of TOF. Several studies, from candidate gene approaches (5) to whole-exome sequencing (WES) studies (6, 7), demonstrate that the genetic landscape of TOF is heterogeneous. Rare chromosomal anomalies are commonly associated with TOF, particularly for syndromic TOF. The 2 most frequently observed cytogenetic aberrations — trisomy 21 (Down syndrome) and 22q11.2 deletion (DiGeorge syndrome) — are associated with TOF in 7% and 15% of cases, respectively (8, 9). Additionally, WNT signaling pathway genes spanned by rare deletions are significantly enriched in more than 3% of patients with TOF (10). Rare coding mutations in *TBX1* (11), *JAG1*, and *NOTCH2* (12, 13) are also found in syndromic TOF patients. Some of these genes, along with others such as *NOTCH1* and *FLT4*, are mutated with lower frequency in isolated (nonsyndromic) TOF (13). While genes causing rare Mendelian, mostly syndromic, forms of TOF have been identified, those underlying the majority of isolated TOF (~80%) remain incompletely characterized (14).

A large WES study by the Pediatric Cardiac Genomics Consortium (PCGC) described de novo mutations as well as rare inherited recessive and dominant variants contributing to a range of CHD phenotypes, including TOF (6). In their study of predominantly European (EU) ethnicity, identification of recessive and dominant genotypes was enhanced by accommodating for ethnic background. Interestingly, within defined CHD categories, there are well-described anatomic differences of the proximal outflow tract between different ethnic populations. For example, the ratio of conoseptal versus conoventricular septal defects is markedly increased in East Asian versus European populations. Yet, to date, a thorough comparison of the genetic landscape of TOF among different ethnic populations has not been reported. Here, we report a whole-genome sequencing (WGS) study of 146 nonsyndromic TOF trios of Chinese ethnicity, with a focus on the coding regions, and explore the differences between the Chinese and EU cohorts. In addition to identifying overlaps with the known TOF gene loci, we identify potentially novel gene candidates. By integrating these genetic findings with spatiotemporal analysis of human cardiogenesis, we delineate developmental time points and gene ensembles contributing to the development of TOF in the human heart.

## Results

### Cohort characteristics and sequencing.

We performed WES on 150 trios of nonsyndromic Chinese patients with TOF screened to exclude a 22q11.2 deletion. Among these, 2 trios were found to be biologically unrelated, while 2 patients had trisomy 21. Exclusion of these 4 proband samples resulted in a total of 146 TOF trios for the subsequent analysis. Median sequencing depth for these 146 trios (*n* = 438) was 38.6 times, and in 86.3% of the samples, the depth was greater than 30 times (Supplemental Table 1; supplemental material available online with this article; https://doi.org/10.1172/jci.insight.152198DS1). Demographic and clinical features are reported in Supplemental Table 2.

### Common and ultra-rare variant association analysis in known TOF-associated genes.

First, we explored the association of candidate TOF genes implicated in recent genome-wide association studies (GWAS). For common variants, except rs7863990 at *SMARCA2* originally reported to be associated with TOF from a Chinese GWAS that showed marginal association (odds ratio = 1.56; 95% CI: 0.97–2.44; *P* = 0.066), no significant association was detected in TOF-associated loci reported from European GWAS (Supplemental Figure 1) (15).

Next, we examined ultra-rare variants absent from any public database in 3 genes implicated from prior WES studies, *NOTCH1*, *FLT4*, and *KDR*. All have been previously reported with increased mutational burden in TOF cases of European ancestry (6, 7). We thus examined the overrepresentation of variants in these genes in our Chinese cohort. Among 146 TOF cases, we detected 6 patients with ultra-rare damaging variants in these 3 genes: 2 in *KDR* (1.4%), 2 in *FLT4* (1.4%), and 3 in *NOTCH1* (2.1%). One patient had both *FLT4* and *NOTCH1* variants, accounting for 4.1% of the Chinese TOF cases. In contrast, our control group (*n* = 590) had 13 variants in 12 patients, a rate of 2.0%. These included 3 in *KDR* (0.3%; 1 control with 2 *KDR* variants), 1 in *FLT4* (0.2%), and 9 in *NOTCH1* (1.5%). Thus, the Chinese patients with TOF had a 2-fold increase (4.8% in cases versus 2.0% in controls) in mutational burden in known TOF-associated genes, though no statistically significant association was detected (burden test *P* = 0.146). Of note, the proportion of carriers in the Chinese population was considerably lower than that of the European populations (i.e., 2.3% for *KDR*, 2.3%–5.1% for *FLT4*, 3.5%–4.5% for *NOTCH1*). Overall, we did not detect statistically significant enrichment of ultra-rare or rare (minor allele frequency < 1%) damaging variants surpassing multiple testing on a genome-wide scale, suggesting a larger sample size is needed to more confidently compare the burden of protein truncating or damaging protein-altering variants in the Chinese TOF cohort.

### De novo protein-altering single nucleotide variants and insertion-deletions.

Previous WES studies showed that rare de novo mutations (DNMs) underlie 8%–10% of CHD cases and rare, recessively inherited mutations might additionally account for about 1.8% of patients with CHD (6). Thus, we next focused on these 2 types of rare protein-altering variants with the highest penetrance to evaluate their contribution to TOF across the 2 populations. Among the 146 nonsyndromic Chinese TOF trios, we identified 140 protein-altering DNMs in 92 cases, including 28 loss-of-function (LoF) DNMs (Supplemental Table 3). Compared with the EU subset of the PCGC TOF cohort, our analysis of the Chinese nonsyndromic TOF cohort had a larger proportion of patients with DNMs across all mutational classes (protein-altering DNMs: 63% in Chinese versus 56% in PCGC; LoF DNMs: 17% versus 10%; synonymous DNMs: 33% versus 25%); this is likely due to the more even coverage of coding regions in our WGS Chinese cohort compared with the WES in the EU PCGC cohort. Among these DNMs, 9 DNMs are considered clinically actionable, i.e., pathogenic or likely pathogenic, through strict application of the American College of Medical Genetics and Genomics and the Association for Molecular Pathology (ACMG-AMP) guidelines (Supplemental Table 4). Clinically, this resulted in a genetic diagnostic rate of around 6% (9/146) in our TOF cohort.

**Figure 1:**
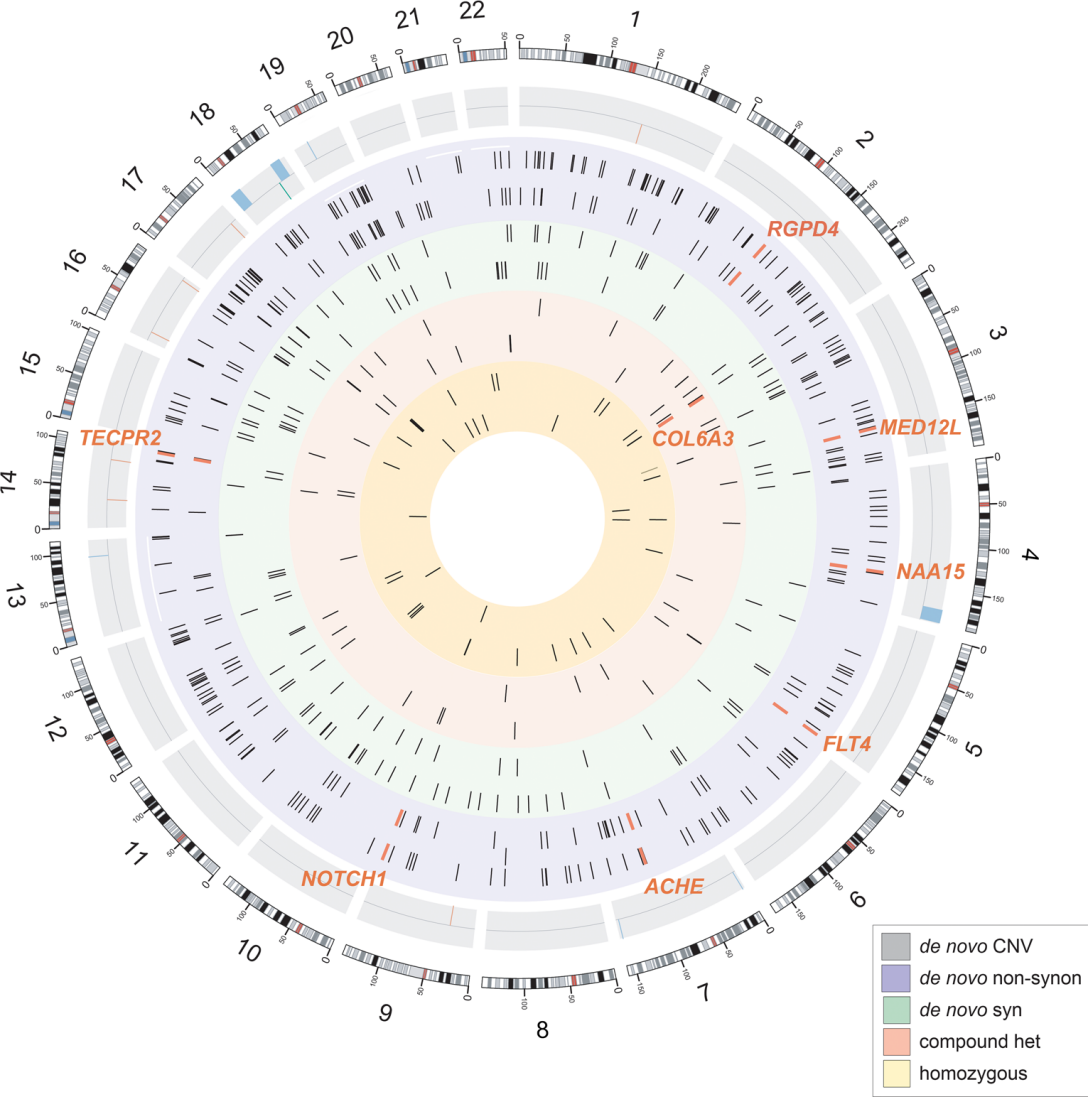
Circos plot of genome-wide variants in Chinese and European patients with TOF. From external to internal tracks: gray circle represents de novo copy number variants found in exonic (outer) and nonexonic regions (inner) regions. Green bar in track 2 represents the only de novo nonexonic duplications. Blue circle represents de novo nonsynonymous variants in European (outer) and Chinese (inner) patients with TOF. Green circle represents de novo synonymous variants in European (outer) and Chinese (inner) patients with TOF. Red circle represents compound heterozygous variants in European (outer) and Chinese (inner) patients with TOF. Yellow circle represents homozygous variants in European (outer) and Chinese (inner) patients with TOF. Orange ticks and labels indicate common genes containing variants in both European and Chinese patients with TOF. See also Supplemental Table 14.

To determine if DNMs contributed significantly to the TOF risk in the Chinese cohort, we assessed the burden of the DNMs in cases relative to the expected control frequencies using denovolyzeR. As was used for WES studies of the EU PCGC cohort, we also used MetaSVM to define damaging missense variants in all subsequent analyses. Similar to the findings of the PCGC, we detected a significant global burden of damaging, protein-altering DNMs (1.5-fold enrichment, *P* = 0.002), particularly LoF DNMs, in patients with TOF (Table 1). These damaging DNMs were largely enriched in genes intolerant to LoF changes (*P* = 4.1 × 10^–4^; *P_LoF_* = 0.004). Given the excess global mutational burden, we next evaluated whether the genes harboring DNMs in TOF cases significantly overlapped with either known human CHD genes or the DNM gene list previously reported in the PCGC CHD (including TOF) cases (6). We observed a significant overlap between known human CHD genes overall and by mutation types (13-fold enrichment for LoF, *P_LoF_* = 3.1 × 10^–4^; 4.8-fold enrichment for damaging DNMs, *P* = 0.004). Excessive damaging DNMs were found exclusively among the CHD genes known to show a dominant effect (*P* = 8.7 × 10^–4^) but not among the known recessive CHD genes. Similarly, we detected significant enrichment of damaging DNMs among genes previously found with protein-altering DNMs in the PCGC CHD cohorts (*P* = 2.1 × 10^–5^). When compared with the overall PCGC CHD cohort, a stronger overlap was observed with the TOF subset (Table 1). The overlap was attenuated but remained significant (*P* = 0.002) after removal of the known CHD genes, implying a substantial phenotypic specificity contributed by these genes. Besides the known CHD genes, 4 genes were found to have DNMs in both Chinese and PCGC European TOF cases but not in control trios comprising parents and unaffected siblings of patients with autism in the Simon Simplex Collection. These included 3 genes (*NAA15*, *RGPD4*, *TECPR2*) with LoF DNMs and 1 gene (*ACHE*) with missense DNMs (Combined Annotation Dependent Depletion [CADD] > 20) in Chinese TOF cases (Figure 1 and Table 2). Interestingly, we did not observe a significant enrichment of DNMs in chromatin-modifying genes related to CHD (*P* > 0.2), and similar enrichment results were obtained when the criterion for damaging was relaxed to CADD more than 20.

### De novo copy number variations.

In addition to single nucleotide variations (SNVs) and insertion-deletions (indels), WGS confidently detects larger copy number variations (CNVs) (>50 bp) based on read depth, split reads, and read pairs (16). We curated a set of high-confidence CNVs using a recently developed machine learning–based method, CNV-JACG, to classify CNV calls from an ensemble of CNV callers (17). In total, we identified 16 (4 large and 12 small) de novo CNVs in 14 patients. The de novo CNV rate of 9.6% (14/146 = 9.6%; 95% CI: 5.8% to 15.5%) in our trios was much higher than that in the general population (usually quoted as <2%) but comparable to the reported rates in TOF cohorts (ranging from 4.6% to 8.0%) (18).

**Figure 2:**
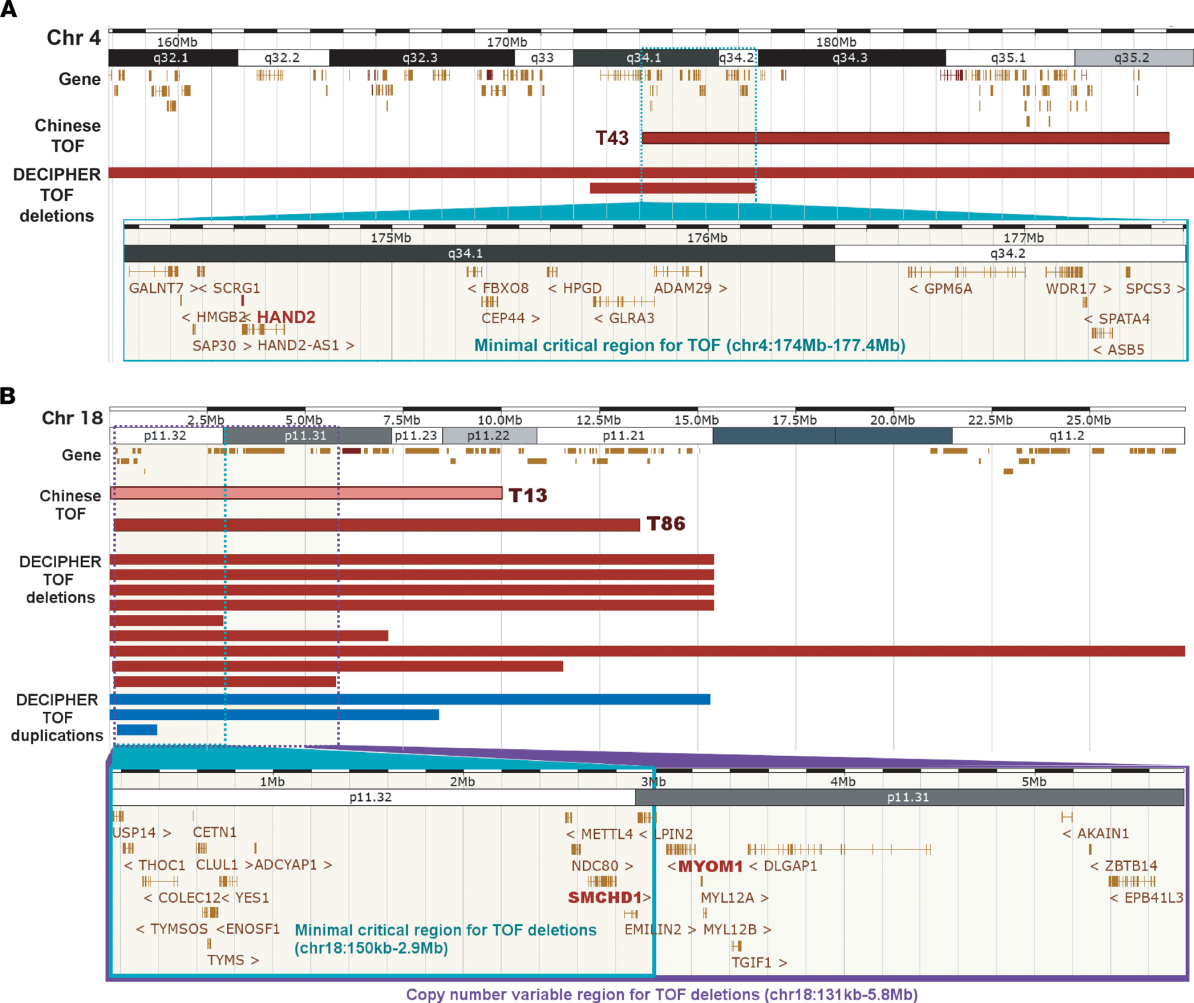
De novo CNVs disrupting coding exons in Chinese patients with TOF. Regions of overlap between the de novo CNVs of Chinese TOF patients and CNVs of DECIPHER TOF patients at (**A**) 4q34 and (**B**) 18p11 are shown (red: deletions; pink: mosaic deletion; blue; duplications). Genes encompassed in the minimal critical regions for TOF deletions are enclosed by the green boxes. Candidate genes prioritized using transcriptomic and epigenomic data from NIH Roadmap Epigenomics Project are bolded. For the 18p11 region, genes in the copy number variable region, denoting the chromosomal location of the shortest DECIPHER TOF deletion having more than 50% reciprocal overlap with Chinese TOF deletions, are shown within the purple box.

Among the 16 de novo CNVs, 8 were genic deletions covering exonic regions (Table 3). To identify candidate TOF genes disrupted by these de novo CNVs, we compared these deletions with those reported for patients with TOF in the DECIPHER database. First, we collapsed the highly overlapping deletions for TOF in DECIPHER into 2 copy number variable regions at 4q34 and 18p11. Compared with an average of approximately 11% across the genome, both regions (28% for 4q34 and 15%–16% for 18p11) had a considerably higher proportion of DECIPHER patients with CHD, which suggests the presence of genes associated with cardiac development in the regions. Second, for the 4q34 locus, a known CHD gene, *HAND2*, was recurrently disrupted by TOF deletions (1 large de novo deletion in Chinese cohort and 2 TOF deletions in DECIPHER database) (Figure 2A) (19). We then used *HAND2* as a benchmark to determine the epigenomic and transcriptomic signatures to further prioritize the TOF-associated genes overlapped by de novo CNVs. Based on data from the NIH Roadmap Epigenomics Project, stronger epigenomic and transcriptomic signals for cardiomyocytes relative to the induced pluripotent stem cells were observed for *HAND2* (Supplemental Figure 2). A similar genomic signature was also observed for *SORBS2*, which was recently suggested to be a CHD-associated gene with a *Sorbs2*^–/–^ mouse mutant that displayed a variety of atrial septal phenotypes (20). Based on these genomic signatures, we further prioritized 3 candidate genes, *SMCHD1*, *MYOM1*, and *PTPRM*, at 18p11 (Figure 2B and Supplemental Figure 3). *SMCHD1* is located within the minimal critical region for TOF and was disrupted by 2 de novo deletions in Chinese patients with TOF and 12 DECIPHER TOF CNVs (9 deletions and 3 duplications; Supplemental Figures 4 and 5). Interestingly, one of the de novo deletions in a Chinese patient with TOF was predicted to be a mosaic deletion according to the read depth and allelic ratio. The patient carries another mosaic deletion at 18q. The mosaic deletion rate of 0.68% (1/146) in our study is comparable to the reported rate of less than 0.5% for detectable mosaicism in peripheral blood (21). *SMCHD1* was also reported to have de novo missense variants in 1 PCGC European TOF patient and 1 non-TOF CHD patient, which strongly supports its relevance in the pathogenesis of TOF (6). Except for these two 18p de novo deletions, we did not detect any other CNVs involving any gene in which a de novo SNV/indel had been identified in our trios. The other 8 de novo noncoding CNVs are listed in Supplemental Table 5.

### Inherited recessive variants.

The PCGC previously reported an increased burden of rare, damaging recessive (homozygous and compound heterozygous) variants in CHD cases compared with controls (6). Using the same criteria (global minor allele frequency [MAF] < 0.001 and predicted damaging by MetaSVM), we detected 33 rare, damaging recessive (11 homozygous and 22 compound heterozygous) genotypes in 28 Chinese TOF cases (Supplemental Table 6). None of these variants mapped to known human or mouse recessive CHD genes. Only *COL6A3* had recessive damaging variants in both Chinese and European PCGC TOF cases. If less stringent filtering criteria of CADD > 20 and MAF < 0.01 were used, we observed 237 recessive (41 homozygous and 196 compound heterozygous) damaging genotypes (Supplemental Table 7), of which 5 genes (*MYOM2*, *LAMB3*, *GOLGA4*, *DNAH7*, and *AHNAK*) were recurrently disrupted in TOF cases but not in pseudocontrols consisting of untransmitted alleles of parents (Supplemental Table 8).

### Enriched functional annotations in Chinese TOF cases.

**Figure 3:**
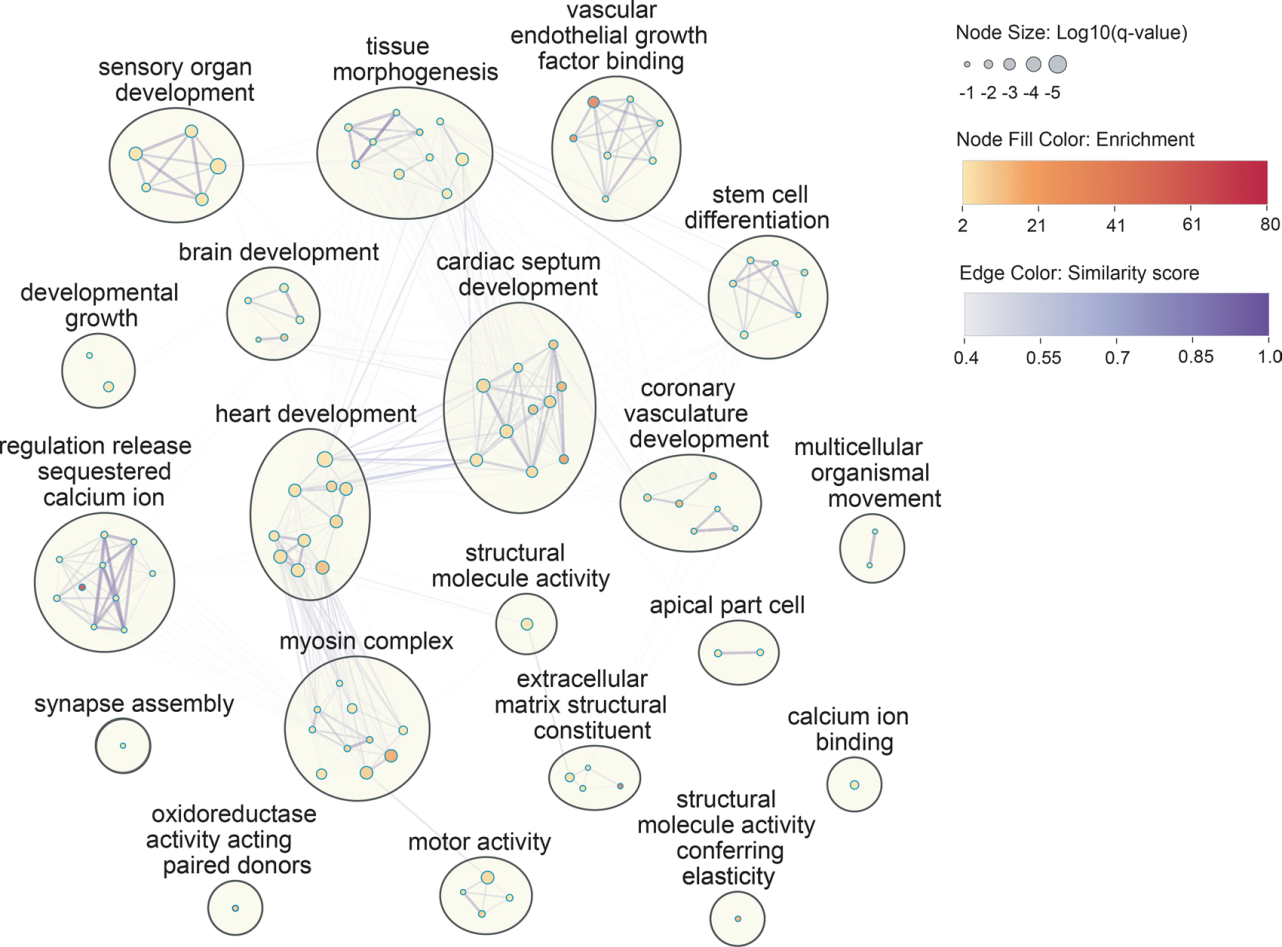
GO cluster network enrichment in candidate genes with damaging variants in 146 Chinese TOF trios. Genes with damaging variants, including all genes with de novo or recessive loss-of-function (LoF) and damaging missense (CADD > 20) variants, and selected candidate genes encompassed by de novo coding deletions were included in the gene set enrichment analysis. The nodes represent GO terms that were statistically significant after multiple-testing correction (FDR) (*q* < 0.1). Similar GO terms (similarity score > 0.3) were grouped as clusters (circles), and within each cluster only the top 10 GO terms with the most significant *q* values are shown (full results in Supplemental Table 9). The size of the nodes represents log_10_(*q* value) of the GO terms. Color gradient of the nodes reflects the enrichment and that of the edges represents the level of similarity between the enriched GO terms.

To probe for mechanistic insights, we performed Gene Ontology (GO) enrichment analysis using the gene list of damaging variants found in Chinese TOF cases. The damaging gene list includes all genes with de novo or recessive damaging (LoF or damaging missense) variants (by CADD > 20) as well as the candidate genes of de novo deletions denoted in Table 3. Enrichment clustering was performed by Metascape (22), which grouped 167 significant GO terms (FDR *q* < 0.1) into a network of 21 clusters. The top 20 clusters were plotted in Figure 3. The least significant cluster can be found in Supplemental Table 9 (cluster 21). The top 5 enriched clusters (*q* < 3.3 × 10^–4^) were represented by the top GO term within each cluster as (i) heart development, (ii) sensory organ development, (iii) cardiac septum development, (iv) motor activity, and (v) myosin complex. The top enriched cluster of heart development contained GO terms related to development of cardiac muscle and morphogenesis, including genes encoding myomesins (*MYOM1* and *MYOM2*) and myosin-related proteins (*MYH2*, *MYH8*, and *MYBPC2*). The third enriched GO cluster represented by cardiac septum development contained GO terms largely related to different aspects of cardiac development and morphogenesis, e.g., cardiac chamber as well as valve and aorta morphogenesis and development. In particular, damaging variants in NOTCH signaling pathways (*q* = 0.005 in heart development and *q* = 0.015 overall) and outflow tract morphogenesis (*q* = 0.09) showed increased burden and grouped within this cluster. This enrichment was partially contributed by LoF DNMs in *NOTCH1*, *JAG1*, and *ROBO1*; in-frame DNMs in *CNTN1*; de novo deletion in *SORBS2*; and homozygous LoF in *RBPJ*. Interestingly, damaging variants were also significantly overrepresented in known TOF-associated vascular endothelial growth factor (VEGF) pathways (lowest *q* = 9.2 × 10^–4^ in the GO enriched cluster) in the Chinese TOF cohort.

### Differentially enriched functional gene sets in Chinese and European TOF cases.

**Figure 4:**
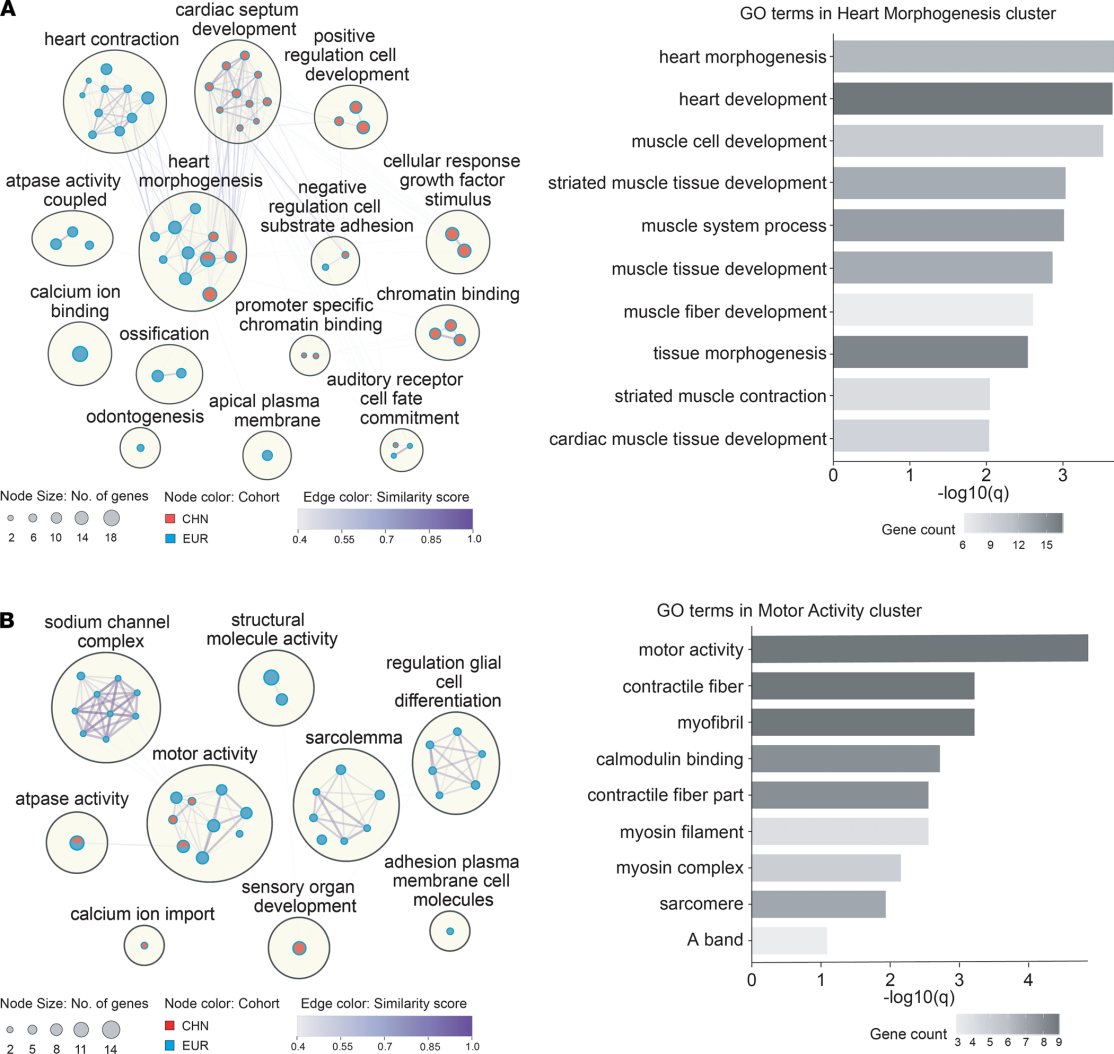
GO cluster network enrichment among the genes. Enrichment among genes with (**A**) de novo and (**B**) recessive damaging variants in Chinese and PCGC EU TOF trios. Color of the nodes reflects the strongest enrichment among the Chinese only (red), European only (blue) or combined (mixed) analyses. Top GO terms of selected clusters showing stronger enrichment in combined analysis are shown on the right panel for de novo and recessive gene lists, respectively. The nodes represent GO terms that were statistically significant after multiple-testing correction (FDR) (*q* < 0.1). Color gradient of the edges connecting the nodes represents the level of similarity between the enriched GO terms.

To examine potential differences between Chinese and European populations, we compared and contrasted our Chinese results with a European cohort, PCGC data extracted for European ethnicity and TOF phenotype. Here, we limited the enrichment analysis to de novo and recessive gene lists and restricted the missense variants to those predicted as damaging by MetaSVM (23). First, as shown in Figure 4, joint analysis of the 2 cohorts resulted in stronger enrichment of 2 core GO clusters represented by heart morphogenesis for de novo and motor activity for recessive gene lists (Supplemental Tables 10 and 11). Top GO terms in both clusters were related to muscle cell development or components of muscle structure, which implied a general overrepresentation of damaging variants in muscle-related genes irrespective of populations or inheritance models (Figure 4, A and B; right panels). Second, in contrast to the predominately muscle-related enrichment in the recessive gene set, enriched terms of the de novo gene set contained diverse cellular components and molecular functions. Notably, we observed differential enrichment of GO clusters between populations, with cardiac septum development uniquely enriched with the de novo gene set in the Chinese patients with TOF, whereas terms involved in heart contraction and sodium channel complex were enriched mainly in European TOF cases.

### DNMs in Chinese TOF cases are enriched in genes differentially expressed in early cardiac progenitors.

**Figure 5:**
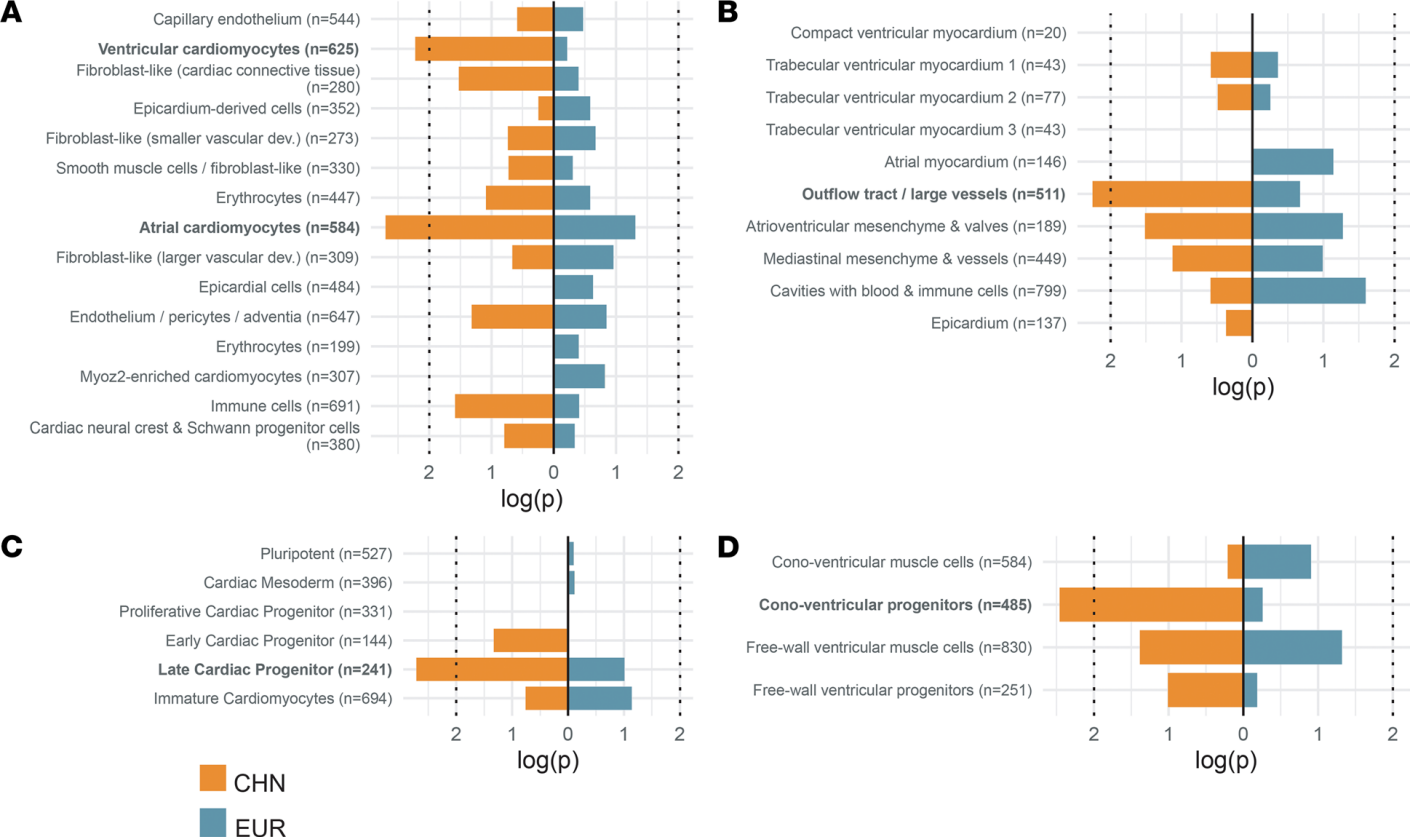
Gene enrichment of de novo damaging variants in cell type clusters implicated in single-cell and spatial transcriptomics. Color of the bars reflects the enrichment among the Chinese only (left; orange) or European only (right; teal) analyses. (**A**) Human embryonic cardiac cell types identified by single-cell RNA sequencing from Figure 3 in Asp et al. (24); (**B**) clusters of the anatomical embryonic cardiac regions of spatial transcriptomic data from Figure 2 in Asp et al. (24); (**C**) cell type clusters identified by single-cell RNA sequencing of in vitro human embryonic stem cell cardiac differentiation in Mononen et al. (25); (**D**) cell type clusters from human embryonic/fetal heart–derived single-cell RNA sequencing in Sahara et al. (26). Cell types with significant enrichment of DNMs (*P* < 0.01; dotted lines) are highlighted in bold. Numbers in parentheses indicate the total number of genes analyzed in each category/cluster.

To determine if specific cardiac cell types were implicated in the DNM gene set, we asked if there was enrichment of DNMs among differentially expressed genes in 4 single-cell RNA-Seq data sets. Two of these were data from a spatial transcriptomics study: 1 data set derived from a single-cell RNA-Seq analysis of a 6.5 to 7 weeks postconception (PCW) human heart, and a second data set derived from a spatial transcriptomics single-cell RNA-Seq analysis of a 6.5–7 PCW human heart. A third data set was from in vitro single-cell profiling of human embryonic stem cell (hESC) cardiac differentiation. A fourth data set was from human embryonic/fetal heart–derived single-cell RNA-Seq analysis between 4–5 and 10 PCW. Separately analyzing these 4 single-cell cardiac data sets, all of which differed somewhat in tissue type and techniques, allowed us to probe mutational enrichments from multiple perspectives (Figure 5).

Among the 15 cell type clusters identified in single-cell RNA-Seq analysis of human embryonic heart (6.5–7.0 PCW) (24), genes differentially expressed in atrial (4.3-fold enrichment; *P* = 1.6 × 10^–3^) and ventricular cardiomyocytes (3.8-fold enrichment; *P* = 5.9 × 10^–3^) were significantly enriched with damaging DNMs found in Chinese, but not in European, TOF (Figure 5A). In contrast with cardiac muscle, no enrichment of DNMs was observed in the cardiac neural crest cell cluster. In particular, genes with damaging DNMs showed significant spatial expression in outflow tract or great vessels, consistent with the anatomic defect in TOF (Figure 5B). To validate the contribution of genes with DNMs to cardiogenesis, we further tested this enrichment on 2 additional single-cell transcriptomic data sets, (i) in vitro single-cell profiling of hESC cardiac differentiation (25) and (ii) human embryonic/fetal heart–derived single-cell transcriptomes (26). An excess of TOF DNM genes was observed exclusively in cardiac, particularly proximal outflow tract (conoventricular), progenitors expressing early cardiogenic markers, such as *PDGFRA* and *ISL1* for Chinese, but not European, data (Figure 5, C and D). Of note, the enrichment in atrial cardiac myocytes likely identified the atrial signal common to all early cardiac myocytes prior to chamber specification. Again, these locations are consistent with the anatomic defect in TOF of proximal outflow tract (conus) migration and positioning.

### Temporal and functional patterns of known and potentially novel TOF genes.

**Figure 6:**
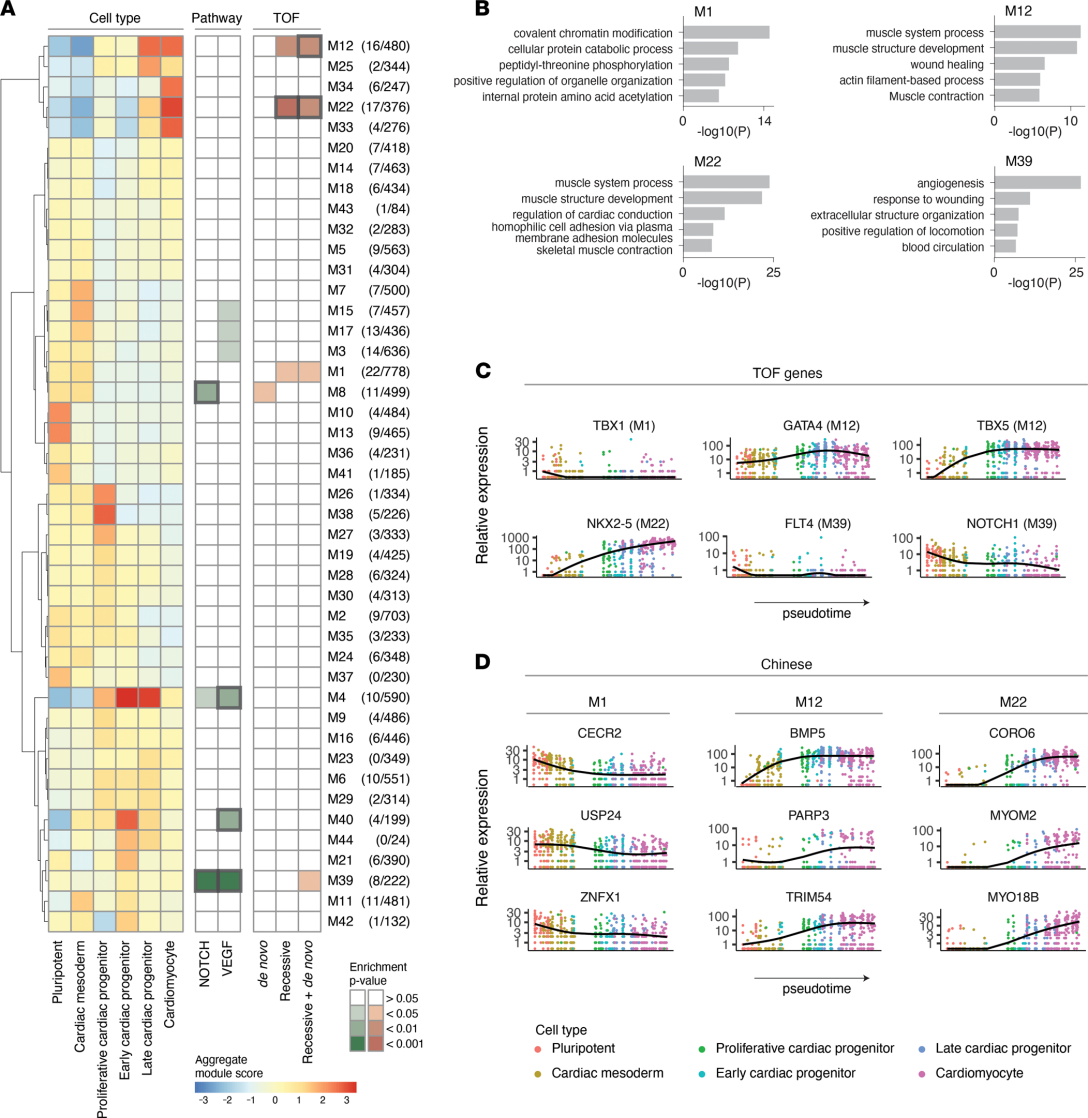
Integration of coexpression modules and signaling pathway enrichment with pseudotime gene expression of genes with de novo and recessive damaging variants in Chinese TOF cases. (**A**) Genes varying across the in vitro cardiac differentiation trajectory grouped into 44 Monocle 3 coexpression modules and visualized as aggregate module scores for developmental states. Some modules exhibited differentiation state-specific aggregation (M10, M13, pluripotency; M38, proliferative cardiac progenitor; M22, M33, cardiac myocyte), with other modules specifically enriched for genes belonging to NOTCH (M8), VEGF (M4, M40), or both NOTCH and VEGF (M39) signaling pathways. In addition, indicated modules were enriched for genes with de novo and recessive damaging variants identified in Chinese TOF cases. The number of genes with damaging variants (total of de novo and recessive) and the total number of genes in each module are shown in parentheses. The bold rectangles in the 2 right panels indicate significance for Benjamini-Hochberg–adjusted *P* value at FDR < 0.10. (**B**) GO analysis of 4 coexpression modules showing enrichment of genes with damaging variants. The top 5 most significant GO terms are shown for each module. (**C**) Pseudotime expression trends of TOF genes over the pseudotime trajectory. The TOF genes are compiled from Morgenthau and Frishman (5), Page et al. (7), and Jin et al. (6). Genes belonging to the modules with enrichment of Chinese TOF candidate genes are shown, and the coexpression modules are indicated in parentheses. (**D**) Pseudotime expression patterns of genes with damaging de novo (CECR2, BMP5, TRIM54, and CORO6) and recessive (USP24, ZNFX1, PARP3, MYOM2, and MYO18B) mutations in Chinese TOF cases. Genes belonging to the coexpression modules with overall enrichment of damaging variants and differentiation stage-specific patterns, e.g., M1, M12, and M22, are shown.

To infer the timing of gene activity implicated in Chinese TOF cases along hESC cardiac differentiation, we performed pseudotime analysis using Monocle 3, which uses an unsupervised algorithm to reveal gene expression kinetics over time in single-cell resolution (27–29). We built a pseudotime trajectory of the cardiac differentiation process (Supplemental Figure 6), curated 44 gene coexpression modules using Louvain method for community detection (30), and converged the modules with differentiation states from pluripotency to cardiomyocytes (Figure 6A). This analysis was performed using all genes in the single-cell data set. Previous studies implicate both *NOTCH* and *VEGF* mutations with cardiac outflow tract anomalies (31); this finding was further supported by enrichment of damaging mutations in *NOTCH* and *VEGF* genes in the Chinese cohort. Hence, we calculated the overall enrichment of *NOTCH* and *VEGF* genes in the coexpression modules.

Analysis of damaging gene variants in each module showed that genes with damaging DNMs were uniquely enriched in M8 (hypergeometric *P* = 0.0170), a module with an early developmental pattern (expressed only in precardiac stages) with enrichment for NOTCH genes (*P* = 0.00131). In contrast, genes with recessive mutations were enriched in M1 (*P* = 0.0487), M12 (*P* = 0.00899), and M22 (*P* = 0.000409), modules showing both early and late developmental patterns but no enrichment for *NOTCH* or *VEGF* pathways. Combinations of genes with de novo and recessive variants resulted in enrichment in an additional module, M39 (*P* = 0.0326), showing a transient pattern over the differentiation states and enrichment for both *NOTCH* (*P* = 6.9 × 10^–5^) and *VEGF* (*P* = 0.000402) pathways (Supplemental Table 13).

We next performed GO analysis using Metascape (22) to infer cellular functions related to the coexpression modules with TOF candidate gene enrichment (Figure 6B). The modules with early developmental patterns, M1 and M8, showed enrichment of terms related to regulation of embryonic development and chromatin modification, whereas late-pattern M12 and M22 were enriched for muscle system process and development-related terms. The temporally transitional module M39 showed enrichment of terms related to angiogenesis, endothelium development, and regulation of the *NOTCH* signaling pathway.

The candidate genes identified in the Chinese cohort belonging to the early-pattern M1 included *KMT2D*, which encodes a histone modifier associated with CHD in Kabuki syndrome (32). Additionally, 3 other genes associated with syndromic forms of CHD (*EVC*, *TEP1*, and *CECR2*) (33–35) were found in the enriched modules with early expression patterns, i.e., M1 and M8. Among the candidate genes with late expression pattern belonging to M12 and M22, several were related to cardiac muscle development (*RYR2*, *XIRP2*, *TTN*, *BMP5*, and *MYO18B*). Furthermore, genes associated with actin-binding (*SYNPO2*, *XIRP2*, and *CORO6*) and microtubule-binding (*KIF26B* and *MAP1A*) properties were identified in these 2 modules.

Several genes previously implicated in TOF were found in coexpression modules showing enrichment for genes with de novo and recessive variants in Chinese TOF cases (Figure 6C). DiGeorge syndrome–associated transcription factor TBX1 is a member of M1, convergent with pluripotent and early mesoderm populations, whereas the cardiac transcription factors *GATA4*, *TBX5*, and *NKX2-5* were found in modules M12 and M22, correlating with cardiomyocyte differentiation. The only module containing significant NOTCH and VEGF signals both was module M39, containing the angiogenesis-related and signaling pathway–related genes *FLT1*, *FLT4*, and *NOTCH1*. We visualized the expression patterns of these known TOF genes along pseudotemporal trajectories of hESC cardiogenesis, correlating expression kinetics with the temporal patterns of coexpression modules. To further assess the temporal expression patterns of the potentially novel TOF genes with de novo and recessive mutations, we visualized these potentially novel candidate genes from the coexpression modules M1, M12, and M22 showing strong early (*CECR2*, *USP24*, and *ZNFX1*) and late (*BMP5*, *PARP3*, *TRIM54*, *CORO6*, *MYOM2*, and *MYO18B*) temporal expression patterns (Figure 6D).

**Figure 7:**
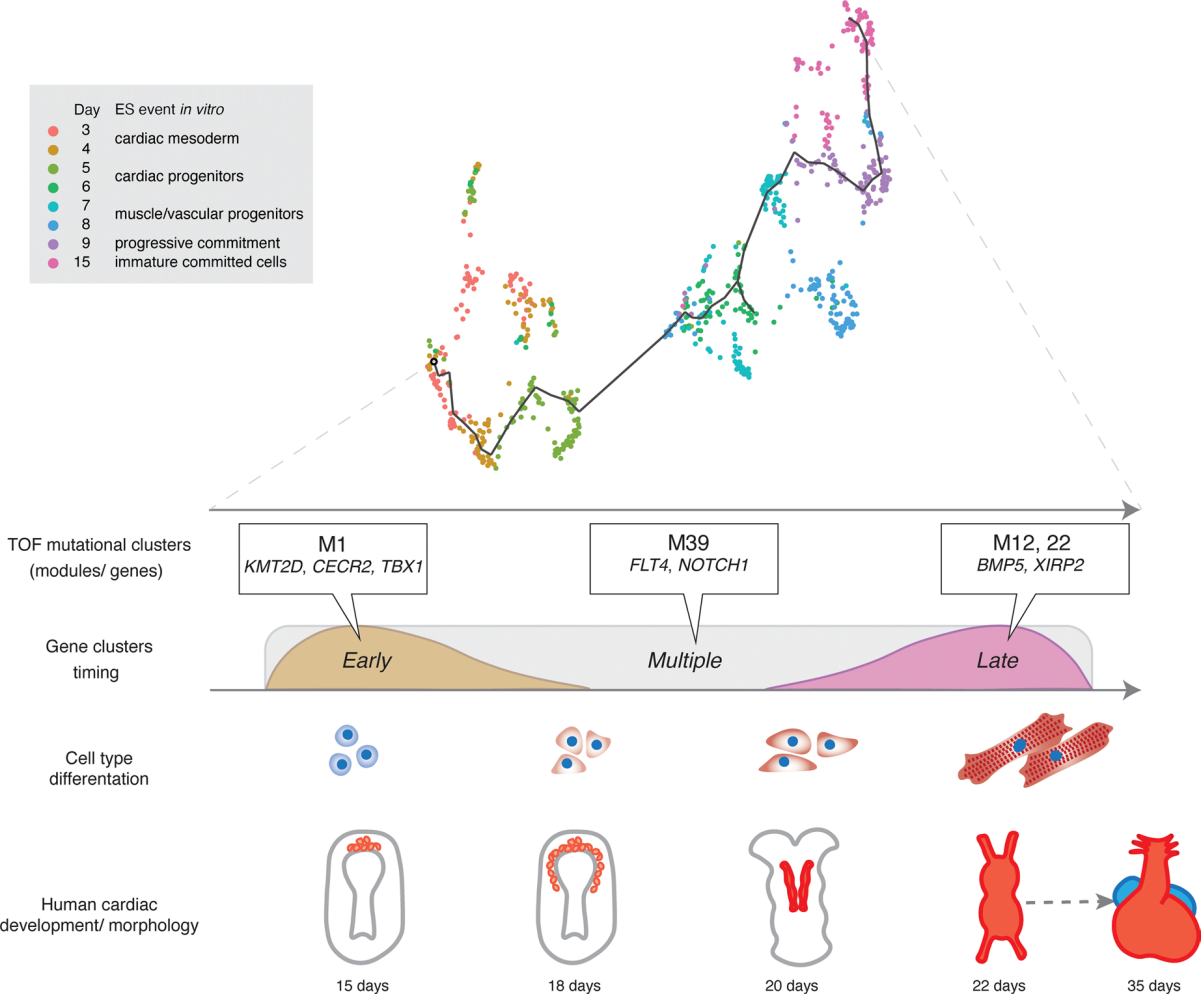
The genetic heterogeneity of TOF is reflected in a diversity of genetic modules, signaling pathways, and developmental timing of effect. Intersection of human cardiac myocardial cell differentiation single-cell RNA-sequencing data set (top) with the mutational landscape of large cohorts of patients with TOF enabled identification of specific genes and gene modules significantly associated with distinct developmental time points (middle). Some genes such as *FLT4* or *NOTCH1* identified as having damaging variants in multiple sequencing studies are expressed at multiple developmental time points, whereas others are significantly constrained to either early (e.g., *KMT2D*) or late (e.g., *BMP5*) time points. Combining sequencing data with single-cell data sets provides new insight into the developmental mechanisms underlying the pathogenesis of TOF.

Taken together, our analysis reveals the developmental expression kinetics of both known and likely novel TOF genes, associating specific genes with discrete developmental pathways and cellular functions (Figure 7). The subsets of the coexpressed candidate genes can be found in Supplemental Table 13.

## Discussion

Our study represents the first large WGS survey of TOF patients of non-European origin to our knowledge. We identified protein-altering DNMs in 63% and LoF DNMs in 17% of patients. Furthermore, we explored genetic landscapes of Chinese and European TOF cohorts by comparing our analysis with the WES of a large TOF cohort of European ethnicity by the PCGC (6). To elucidate the potential impact of damaging rare inherited and de novo variants on the transcriptional regulation of early cardiac populations, we intersected the genetic data with single-cell RNA-Seq analyses of early human heart development.

Our analysis of rare inherited recessive and de novo damaging mutations using GO enrichment analysis suggests that mechanistically many genes were associated with the GO categories of heart development, cardiac septum development, motor activity, and myosin complex. In particular, within the GO cluster related to heart development, NOTCH signaling pathway and outflow tract morphogenesis were among the most significant terms, marked by genes such as *NOTCH1*, *JAG1*, *CNTN1*, *ROBO1*, and *SORBS2*. Our findings further support the important role of NOTCH signaling in the pathogenesis of outflow tract cardiac defects (7, 31) but additionally suggest that mechanisms specific to early heart development are also important in the pathogenesis of TOF.

Ethnic differences in the prevalence of different phenotypes among patients with CHDs may be reflected in the underlying genetic landscape (1, 8). Previous studies comparing Asians and Europeans show a difference in incidence and character between left- versus right-sided heart phenotypes in patients with complex CHDs (36, 37). Similarly, comparison between our TOF results with a Chinese cohort and EU PCGC TOF data showed differential enrichment of GO terms between populations, with cardiac septum development uniquely enriched in the de novo gene set of the Chinese patients with TOF, whereas GO terms involved in heart contraction and sodium channel complex were enriched among the European TOF cases. Although we were limited in power to further explore these differences, to our knowledge this is the first demonstration of functional differences in de novo variants associated with distinct ethnic populations of patients with CHD. Understanding additional factors, such as the environment, in a larger cohort will be important to extend and further explore these findings. Other genetic factors, such as variations in genetic predisposition as well as variable degree of modified penetrance of rare coding variants by common cis-regulatory variants, may also contribute to the variable penetrance of de novo variants and disease expressivity. Last, the relatively low incidence of extracardiac structural malformations (2%) in the Chinese cohort may partially explain a portion of the differences, including the lack of enrichment of chromatin-modifying genes.

To explore the developmental underpinnings of TOF, we intersected the Chinese DNMs with single-cell transcriptional analysis of spatial and temporal aspects of early human cardiogenesis. Among early cell populations of the developing human heart at 6.5–7 PCW, we identified a significant enrichment within genes differentially expressed in atrial and ventricular cardiomyocytes. Intriguingly, similar analysis with DNMs identified in European patients with TOF did not lead to statistically significant findings, suggesting a potential diversity of pathogenetic routes to the final anatomic phenotype of TOF. Intersection with spatial transcriptomics further revealed that genes with DNMs in Chinese patients were specifically expressed in the outflow tract or the large vessels, supporting the embryological hypotheses of aberrant positioning of the proximal outflow tract in TOF; we did not find evidence or enrichment of the neural crest. We also found significant expression of genes with DNMs in a population of conoventricular progenitor cells that populate the proximal outflow tract; this population may play roles in building and guiding the myogenic and nonmyogenic components of the outflow tract (26).

Understanding the early developmental trajectory of cardiac progenitors may be valuable for understanding congenital heart disease. Migration of the second heart field progenitors to the elongating outflow tract peaks between Carnegie stage (CS) 10 and CS14, or 22–35 dpc in human heart development (38, 39), and the currently available human single-cell RNA-Seq data capture only the final days of this process. Assessment of hESC cardiac differentiation overcomes the restricted availability of pre-4.5-week human embryonic single-cell RNA-Seq data. Although lacking the multidimensional context of cardiogenesis, the hESC cardiac differentiation model system recapitulates many important aspects of cardiac differentiation, including the temporal shifts of gene regulatory programs (40, 41).

In further experiments, we intersected genes harboring damaging de novo and recessive variants in our Chinese TOF cohort with modules of coregulated genes in cardiac differentiation, revealing 3 prominent patterns of candidate gene expression: (i) early precardiac commitment stages, (ii) later developmental yet early after cardiac commitment, and (iii) genes coexpressed with *NOTCH* and *VEGF*. The candidate genes following the early expression pattern were coexpressed with the DiGeorge syndrome–associated *TBX1* (42, 43), whereas those with the late expression pattern were coexpressed with *GATA4*, *NKX2-5*, and *TBX5* cardiac kernel transcription factors previously implicated in TOF (44–47). A smaller group of pathogenic genes were enriched in a module specific for NOTCH and VEGF signaling, containing *FLT4* and *NOTCH1* genes, which have been previously reported in large TOF cohorts (6, 7). As a caveat, early gene expression patterns could not be captured in the available human/embryonic fetal single-cell data, which do not cover these early events. Taken together, the coincident enrichment within specific modules suggests that TOF and congenital heart disease cannot be associated with a single process. Rather, the pathogenesis of TOF is associated with multiple processes, in multiple cell types, at multiple time points, acting in both early and late cardiac differentiation. In the light of our data, disturbances in cellular processes may converge to perturb the contribution of the cardiac progenitors to the myogenic component of the proximal outflow tract. Further advances in the in vitro culture of primate embryos may provide a powerful tool for modeling human CHDs (48).

Combined with previous work, our study suggests that additional genome-wide sequencing analyses of CHD patient cohorts with thoroughly characterized clinical phenotypes of distinct ethnicities will be required to fully understand the genetic architecture of TOF and other complex CHD phenotypes. A comprehensive understanding of the genetic basis of CHDs allows identification of developmental time points and events implicated in the onset of CHDs during early cardiogenesis and is central to the rational development of novel therapeutic and diagnostic interventions.

## Methods

### Patients.

A total of 150 trios (150 TOF probands and 300 parents; *n* = 450) were recruited from Shanghai Children’s Medical Center, China. No patients had 22q11 deletion (non–DiGeorge syndrome), with 2% harboring extracardiac structural malformations. DNA was prepared from blood samples at Novogene, and sequencing libraries were constructed and sequenced using Illumina HiSeq X Ten (Novogene). Based on the processed WGS data, 2 cases were molecularly diagnosed with trisomy 21 (Down syndrome) and were thus removed. In addition, 2 parents were found to be not biologically related to the corresponding proband. As a result, 146 trios were considered for further analysis.

### PCGC EU TOF cohort.

We analyzed the de novo and recessively inherited variants reported for the 300 EU TOF trios of the PCGC cohort. TOF patients were defined by “TETRALOGY OF FALLOT” or “DYSPLASTIC (ABSENT) PULMONARY VALVE SYNDROME (TOF WITH APVS)” in Supplemental Table 1 of Jin et al. (6).

### Controls.

We ascertained 590 controls, including 148 pseudocontrols and 442 in-house controls of Chinese ethnicity, for comparison of variant-based and gene-based mutation frequencies. Pseudocontrols comprised the untransmitted alleles of parents of each patient with TOF (including parents of the 2 TOF patients with Down syndrome) and were genetically unrelated to the patients. WGS data of 442 in-house Chinese controls were ascertained from the controls of a population-based WGS study on Hirschsprung disease. The sequencing data were processed using the same data processing pipeline, and variants were joint called together with TOF samples (49).

### WGS data processing and variant calling.

Raw sequence reads (150 bp) were first aligned to human reference genome (hg19) using Burrows-Wheeler Aligner. Aligned reads were then processed according to Genome Analysis Toolkit (GATK) Best Practices recommendations. In brief, Picard was used for duplicate removal, and GATK was used for indel realignment and base quality score recalibration. Small variants, both SNVs and indels, were called by GATK HaplotypeCaller, and variant-based quality control was carried out using GATK variant quality score recalibration (VQSR). VQSR sensitivity tranches of 99.6 and 99.0 were chosen for SNVs and indels, respectively. Additional hard-filtering quality controls were further applied using KGGseq, which excluded variants with sequencing quality score < 30, with mapping quality score < 20, with violation of Hardy-Weinberg equilibrium (*P* < 1 × 10^–5^ in parents), or with missing rate > 10%. Genotypes with genotype quality < 20 or depth of coverage < 8 were classified as missing genotypes. CNVs larger than 50 bp were called using 4 callers to generate a comprehensive CNV call set: (i) CNVnator, (ii) DELLY, (iii) LUMPY, and (iv) Seeksv. For CNVnator, the bin size for genome partition was set to 50 bp. For the other 3 tools, the default parameters were used. The CNV call set was then curated using our in-house-developed machine learning–based curation tools, CNV-JACG, to identify true deletions and duplications (17).

Variant call format (VCF) files are publicly available and have been deposited here: https://www.ldsrc.hku.hk/downloads; password: 235711.

### Annotation.

For small variants, annotation was carried out using KGGseq for protein function with reference to UCSC RefGene transcripts, in silico damaging prediction, and population frequencies in 1000 Genomes Project, ExAC, and gnomAD. LoF rare variants were defined as nonsense, splicing, and frameshift variants with MAF less than 1%. Damaging recessive variants were evaluated using 2 in silico prediction algorithms, MetaSVM and CADD. To compare the enrichment of DNMs across variant classes for Chinese versus PCGC EU TOF data using denovolyzeR, damaging de novo missense variants included only those predicted as damaging by MetaSVM. CNVs were annotated against DGV and DECIPHER v11.0 databases.

### Variant prioritization.

Protein-altering, rare small variants were further considered with regard to the inheritance pattern within each trio.

Ultra-rare damaging variants were defined by being (i) absent in any public database, including 1000 Genomes Project, ExAC, and gnomAD; (ii) absent in 443 patients with congenital gastrointestinal disorder but without TOF; and (iii) LoF variants (stopgain, frameshift, or splicing) or missense variants predicted to be damaging (CADD > 20).

De novo SNVs and indels were defined with the following criteria: (i) present only in the child but absent in both parents, (ii) alternative allele frequency less than 1% in aforementioned public databases on population frequencies, and (iii) minimum alternative allele ratio of 25% in heterozygotes in the proband. All de novo small variants were validated by Sanger sequencing.

CNVs with more than 50% reciprocal overlap were considered highly overlapping and may therefore have similar functional consequence. De novo CNVs were defined as CNVs (i) present in the proband, (ii) absent in both of the parents, and (iii) not highly overlapped with any DGV gold standard CNVs identified in the normal populations. Those de novo CNVs intersecting exonic regions and with size greater than 250 bp and less than 1 Mb were validated by PCR, followed by Sanger sequencing. To identify TOF-associated regions recurrently disrupted by CNVs, copy number variable region for TOF was defined as the chromosomal location of the shortest DECIPHER TOF deletion having more than 50% reciprocal overlap with all the Chinese TOF deletions. To further narrow down TOF-associated genes, the minimal critical region for TOF was defined as the minimal overlapping chromosomal location for the same type of CNVs (deletions/duplications) of Chinese and DECIPHER TOF patients. DECIPHER patients with CHDs were classified by their cardiac phenotypes and abnormalities of the cardiovascular system morphology.

Rare inherited recessive variants were identified with the following criteria: (i) present in heterozygous form in both parents but homozygous alternative in the probands, (ii) alternative allele frequency less than 1% in public databases, (iii) minimum alternative allele ratio of 25% in heterozygotes in both parents, and (iv) minimum alternative allele ratio of 75% in the proband.

The VCF file of the 146 Chinese TOF trios is available at https://www.ldsrc.hku.hk/downloads and follows the gnomAD format: (i) counts of alternative allele, (ii) number of homozygous genotypes, and (iii) number of nonmissing genotypes for each variant in the coding region are included for cases and controls, respectively.

### Genome-wide association analysis of common variants.

Common variant association analysis was performed on 146 patients with TOF and 590 controls of Chinese ethnicity. A total of 7,296,750 common variants (i) with MAF > 0.01, (ii) not violating Hardy-Weinberg equilibrium (*P* > 1 × 10^–4^), and (iii) with missing rate < 5% were tested for association with TOF using logistic regression while adjusting for 3 principal components.

### Clinically actionable variations.

We followed the criteria specified in Alankarage et al. (50) to identify clinically actionable variants for TOF. Protein-altering variants within the 107 genes in the curated actionable CHD gene panel (http://chdgene.victorchang.edu.au) were annotated against known variants in ClinVar database and evaluated for pathogenicity according to the ACMG-AMP guidelines.

### Mutational model.

As described previously, we used the trinucleotide sequence context to derive the expected probability of observing a de novo variant in each gene (51). We considered all protein-coding transcripts in UCSC RefGene. For each base in the coding region, we determined the probability of observing each of the 3 possible single nucleotide substitutions according to the baseline mutation rate provided by gnomAD v2.1.1 while considering the effect of methylation status (52). Similar to our analysis pipeline, the most damaging annotation by KGGseq across all transcripts was considered the coding consequence for each of the possible single nucleotide substitutions. For each gene, the expected probability of mutation was summed for each variant class (synonymous, missense, damaging missense, nonsense, essential splice site, frameshift, start loss, and stop loss). In order to directly compare the de novo mutation enrichment against PCGC data, each deleterious missense variant was predicted using MetaSVM. The probability of frameshift mutation was defined by multiplying the probability of nonsense mutation by 1.25 for each gene. Unlike exome sequencing, the coverage was relatively even across the genome for the WGS data; therefore, we did not adjust for the read depth. As variants with missingness more than 10% were excluded in the variant calling pipeline and could not contribute to de novo variant calling, we assigned the probability of de novo mutation to 0 for sites with more than 10% of samples covered by fewer than 8 sequence reads. In line with the recent model of constraint in gnomAD, no adjustment of divergence score was imposed in our mutational model after the use of the new baseline mutation rate incorporating the effect of methylation. The background gene-based de novo probabilities can be found in Supplemental Table 12.

### Burden of DNMs in patients with TOF.

To determine if there was an increased burden of DNMs overall or in curated gene sets (e.g., known human CHD genes, differentially expressed gene sets, etc.), we tested the enrichment of each class of DNMs based on our mutational model using denovolyzeR (51, 53). We used the same list of known human and mouse CHD genes as in Jin et al. (6). The chromatin gene list was defined as the set of chromatin-modifying genes from Watkins et al. (54). Briefly, observed variant frequencies were compared against expectations in a Poisson framework with reference to the estimated de novo mutation rate in each human gene. While denovolyzeR does not take into account in-frame and stoploss variants, these DNMs were considered for the burden test.

### Burden of ultra-rare damaging variants in patients with TOF.

Combined Multivariate and Collapsing–based burden tests aggregating ultra-rare damaging variants for each or all of the 3 TOF-associated genes (*KDR*, *FLT4*, and *NOTCH1*) were performed for 146 TOF cases against 590 controls using rvtests.

### Functional enrichment analysis.

Gene set functional enrichment analysis enables the identification of biological or molecular functions associated with the disease of interest. Taking into account the potential variations in the haploinsufficiency of the genes with heterozygous and homozygous mutations identified in the TOF trios, we performed separate GO functional enrichment analysis on the sets of genes identified with (i) DNMs, (ii) recessive (including homozygous recessive and compound heterozygous) mutations, and (iii) de novo and/or recessive mutations using Metascape (22). We performed the same analysis on the PCGC EU TOF trios, and enriched functional terms were compared between the 2 cohorts. FDR *q* < 0.1 was considered significant.

### Pseudotime and gene coexpression module analyses.

Modules of coexpressed genes were identified using Monocle 3 package to investigate temporal dynamics of gene expression along the in vitro cardiomyocyte differentiation trajectory. The analysis was carried out according to an online tutorial (55). A Monocle 3 cell data set was generated including 739 cells belonging to pluripotent, cardiac mesoderm, proliferative cardiac progenitor, early cardiac progenitor, late cardiac progenitor, and cardiomyocyte clusters as described in Mononen et al. (25). The reads per kilobase per million mapped reads values were preprocessed using the preprocess_cds function using log normalization, and uniform manifold approximation and projection dimensionality reduction was performed. Pseudotime positions of the cells and the root node of the pseudotime trajectory were determined using learn_graph and order_cells functions. Next, graph_test function was used to identify genes that varied over the pseudotime trajectory, and genes with significant change over pseudotime (*q* < 0.05) were included in the subsequent analyses. Louvain community analysis was performed using the find_gene_modules function including a range of values as an input for the resolution argument in order to choose the modularity with the highest value. Optimization of the resolution argument led to identification of 44 gene coexpression modules. Aggregate module scores were calculated for each differentiation stage using aggregate_gene_expression function, and the aggregate scores were visualized using the pheatmap function. The modules were clustered according to the Ward1 (or ward.D) method, and the hierarchical clustering is indicated as a cluster tree. Metascape was used for GO analysis of specific gene coexpression modules.

### Enrichment of pathways and damaging variants in coexpression modules.

To add information of known TOF-related signaling pathways to the module heatmap, *P* values for overenrichment of NOTCH and VEGF signaling pathways were calculated based on the cumulative distribution function of the hypergeometric distribution. First, pathway gene lists were compiled for *NOTCH* (GO:0007219) and *VEGF* (GO:0005021, GO:0038085, GO:0010573, GO:0043184, GO:0005172, GO:0048010 and GO:0038084) signaling pathways, and the pathway gene lists were intersected with modules. Hypergeometric *P* values, expected number of successes, and fold enrichments were calculated according to the online tool provided by the Graeber Lab (https://systems.crump.ucla.edu/hypergeometric/index.php), and significant *P* values (<0.05) for overenrichment were visualized for each module.

Enrichment of genes with damaging de novo and recessive variants in Chinese TOF cases was calculated in the same manner as it was calculated for signaling pathways based on the hypergeometric distribution. All genes with damaging DNMs, excluding synonymous variants and damaging recessive variants with CADD > 20, were included in the analysis.

### Pseudotime expression patterns of known and potentially novel TOF genes.

Several known TOF-associated genes belonged to the coexpression modules showing enrichment of the likely novel TOF candidate genes. The pseudotime expression patterns of these known TOF genes were plotted using the plot_genes_in_pseudotime function in Monocle 3.

Regression analysis was performed according to the Monocle 3 tutorial (55) on the potentially novel TOF candidate genes belonging to modules M1, M12, and M22 to identify the candidate genes with strongest association with pseudotime, i.e., the genes with the steepest coefficient. Following the online tutorial for regression analysis, fit_models and coefficient_table functions were used to calculate change of each gene over the pseudotime under Wald’s test. The method adjusts the *P* value for multiple testing using the method of Benjamini and Hochberg. The genes with strongest association to the pseudotime were plotted using the plot_genes_in_pseudotime function.

### Statistics.

For functional enrichment analysis using Metascape and coexpression module enrichment, *P* values were determined by hypergeometric distribution, and *q* values were obtained by Benjamini and Hochberg FDR. For pseudotime analyses, Wald’s test was used, and adjusted *P* values were obtained by FDR. Unless otherwise noted in the text, a *P* value of less than 0.05 was assigned significance.

### Study approval.

The study was performed in accordance with the ethical principles of the Declaration of Helsinki. Written informed patient consents were obtained and the study was performed in agreement with the approved guidelines of The Ethics Committee of Shanghai Children’s Medical Centre affiliated with Shanghai Jiaotong University School of Medicine, Shanghai, China.

## Author contributions

KRC designed, directed, and obtained initial funding for the project. CSMT, MM, and HH contributed equally to this work. CSMT and WYL performed variant and functional enrichment analyses. MM performed the pseudotime analysis with input from MS, EE, KRC, and PJG. HH performed the clinical investigations, phenotypic characterization, sample recruitment, sequencing data management, and variant validation. CSMT, MM, SCJ, XZ, WYL, YY, HH, and PJG analyzed and interpreted the data. CSMT, MM, and PJG wrote the manuscript with input from WYL, SCJ, XZ, MMGB, QL, YY, MS, EE, KRC, HH, and PKHT. PKHT, KRC, MMGB, and PJG designed and supervised the study and acquired funding.

## Supplementary Material

Supplemental data

Supplemental tables 1-14

## Figures and Tables

**Table 1:**
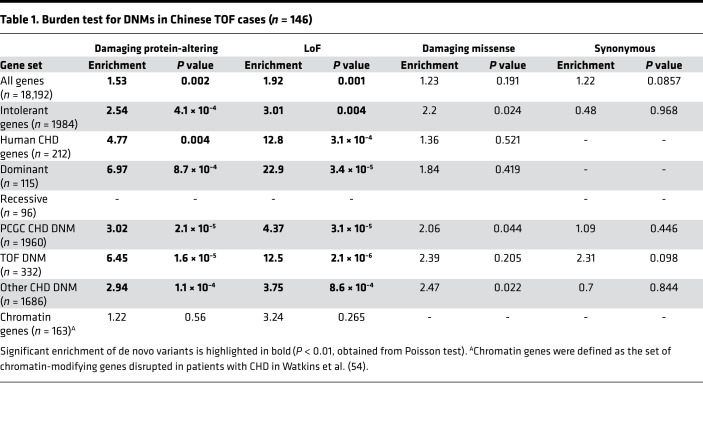
Burden test for DNMs in Chinese TOF cases (*n* = 146)

**Table 2:**
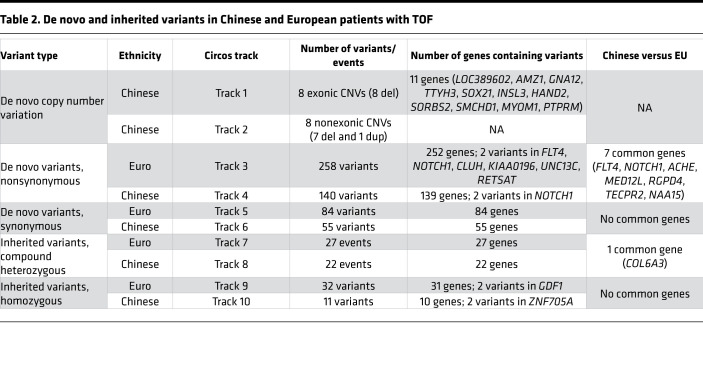
De novo and inherited variants in Chinese and European patients with TOF

**Table 3:**
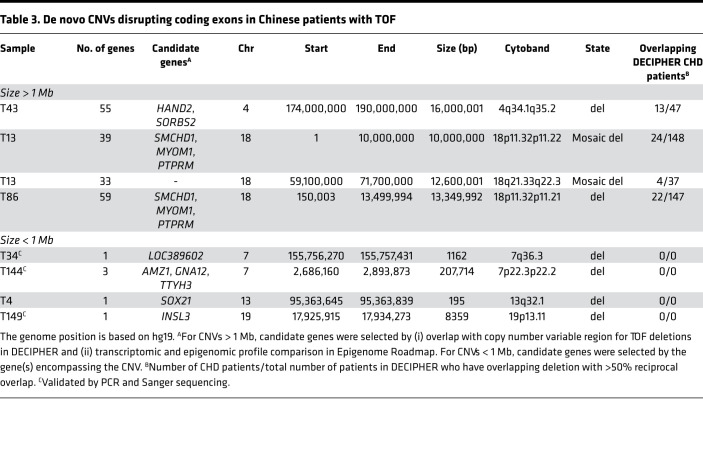
De novo CNVs disrupting coding exons in Chinese patients with TOF

## Version 1 12/14/2021

In-Press Preview

## Version 2 01/25/2022

Electronic publication
