# Supplementary material for: Sequencing of a Chinese tetralogy of Fallot cohort reveals clustering mutations in myogenic heart progenitors
Source: JCI Insight. 2022 Jan 25;7(2):e152198. doi: 10.1172/jci.insight.152198 (PMC8855809; doi:10.1172/jci.insight.152198)
Supplement: Supplemental data [file jciinsight-7-152198-s011.pdf]

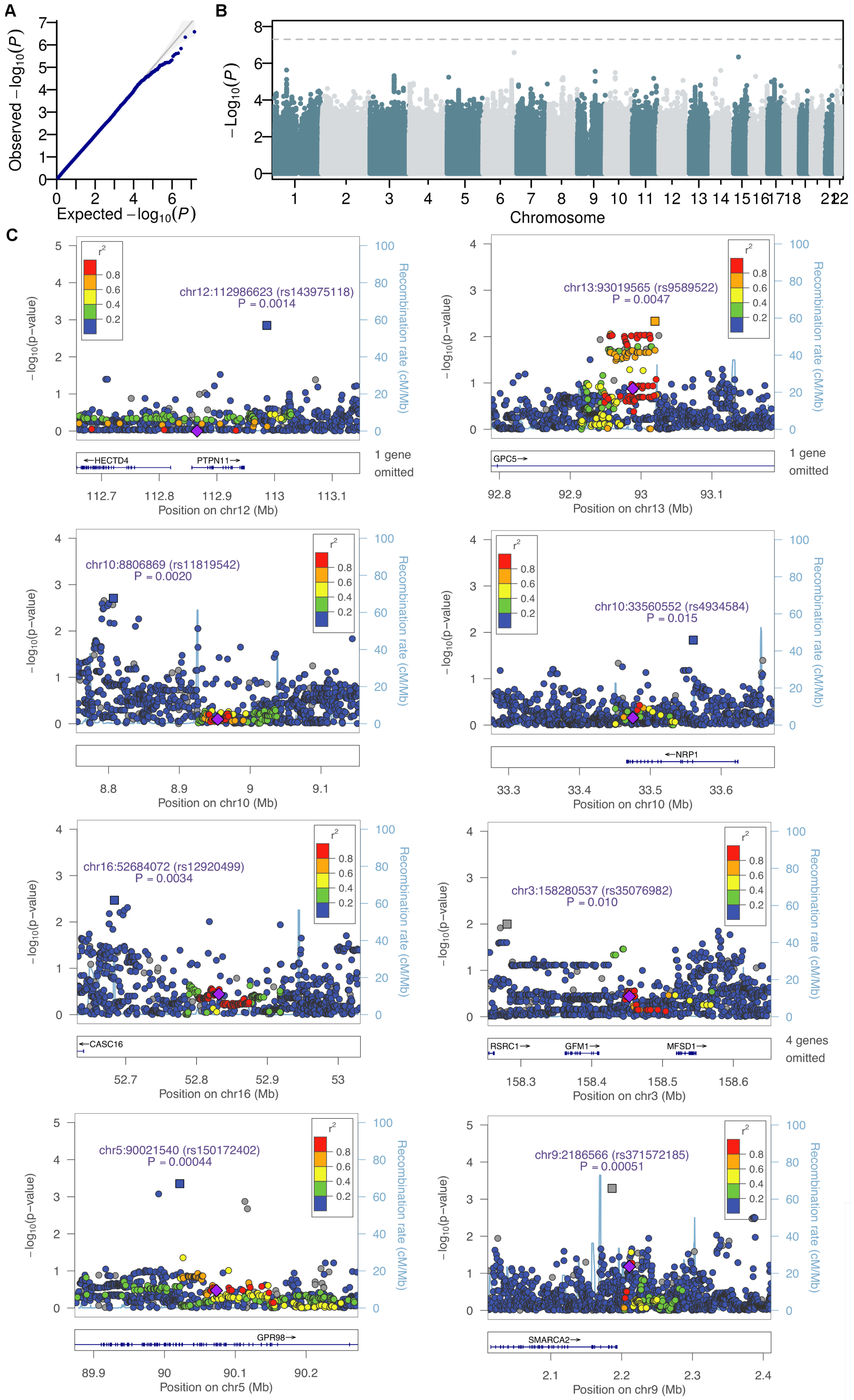

A
B
C
Observed −log10(P)
Expected −log10(P)
−Log10(P)
Chromosome
chr12:112986623 (rs143975118)
P = 0.0014
HECTD4
PTPN11
1 gene omitted
Position on chr12 (Mb)
chr13:93019565 (rs9589522)
P = 0.0047
GPC5
1 gene omitted
Position on chr13 (Mb)
chr10:8806869 (rs11819542)
P = 0.0020
Position on chr10 (Mb)
chr10:33560552 (rs4934584)
P = 0.015
NRP1
Position on chr10 (Mb)
chr16:52684072 (rs12920499)
P = 0.0034
CASC16
Position on chr16 (Mb)
chr3:158280537 (rs35076982)
P = 0.010
RSRC1
GFM1
MFSD1
4 genes omitted
Position on chr3 (Mb)
chr5:90021540 (rs150172402)
P = 0.00044
GPR98
Position on chr5 (Mb)
chr9:2186566 (rs371572185)
P = 0.00051
SMARCA2
Position on chr9 (Mb)
−log10(p-value)
Recombination rate (cM/Mb)
r2

Supplementary Figure 1. Association results of genome wide association analysis (GWAS) on common variants. (A) Quantile-quantile plot and (B) Manhattan plot for GWAS. (C) Regional plots of loci previously reported to be associated with TOF (see Supplementary Table x). The TOF-associated SNP reported in previous publication is highlighted by purple diamond. Colour indicates the linkage disequilibrium (LD; r2) with the reported TOF-associated SNP. The lead SNP of each locus is denoted in square. Chromosomal position and significance of association are reported for each lead SNP.

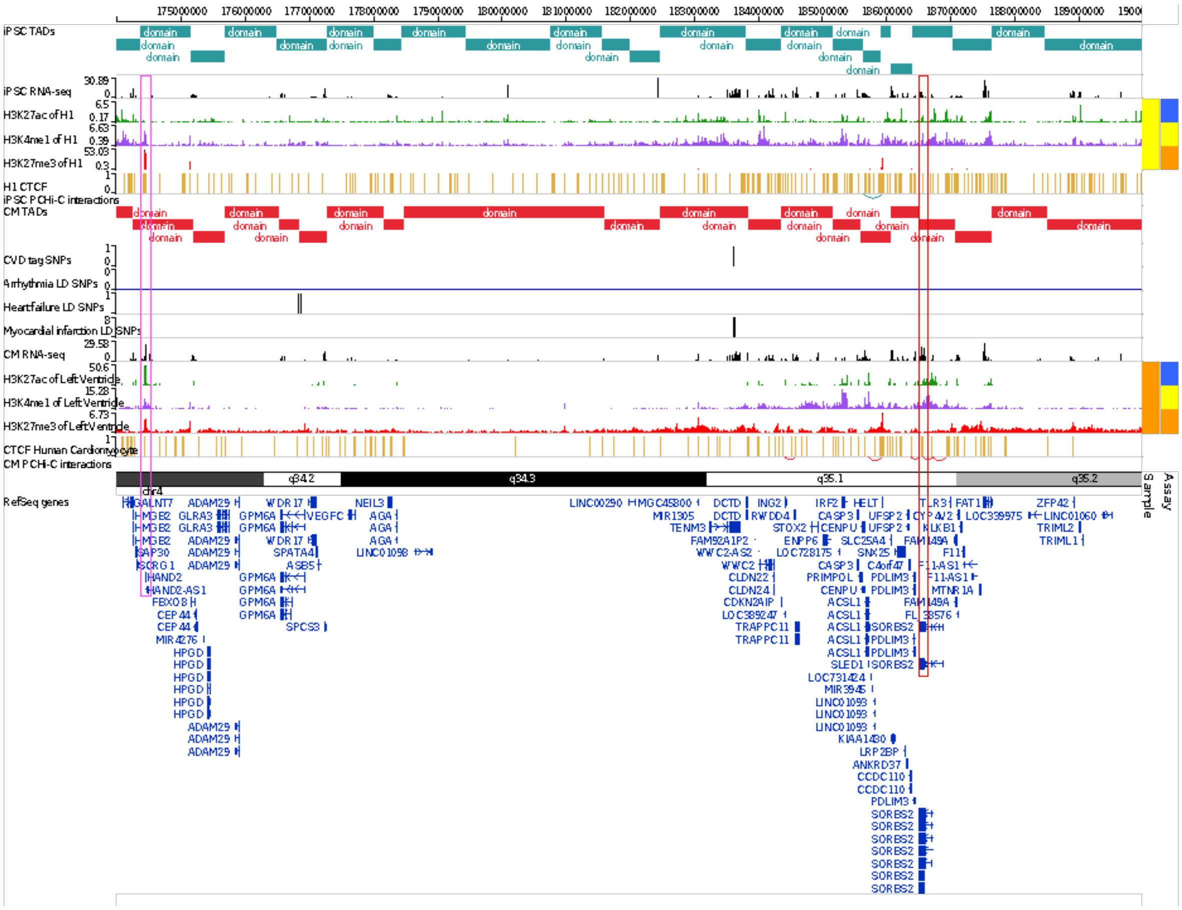

Supplementary Figure 2. Epigenomic landscape of candidate genes in the 4q34.1q35.2 *de novo* deletion locus. Plot of gene expression and epigenomic profiles (H3K27ac, H3K4me1 and H3K27me3) of induced pluripotent stem cells (H1 for iPSC, top) and iPSC-derived cardiomyocytes (left ventricle for CM, bottom) for genes encompassed in the 4q34.1q35.2 *de novo* deletion locus (chr4: 174000000-190000000, hg19) from the Epigenome Roadmap Project/ENCODE using the WashU Epigenome Browser (v46.2). Focus bars for *HAND2* (magenta) and *SORBS2* (red).

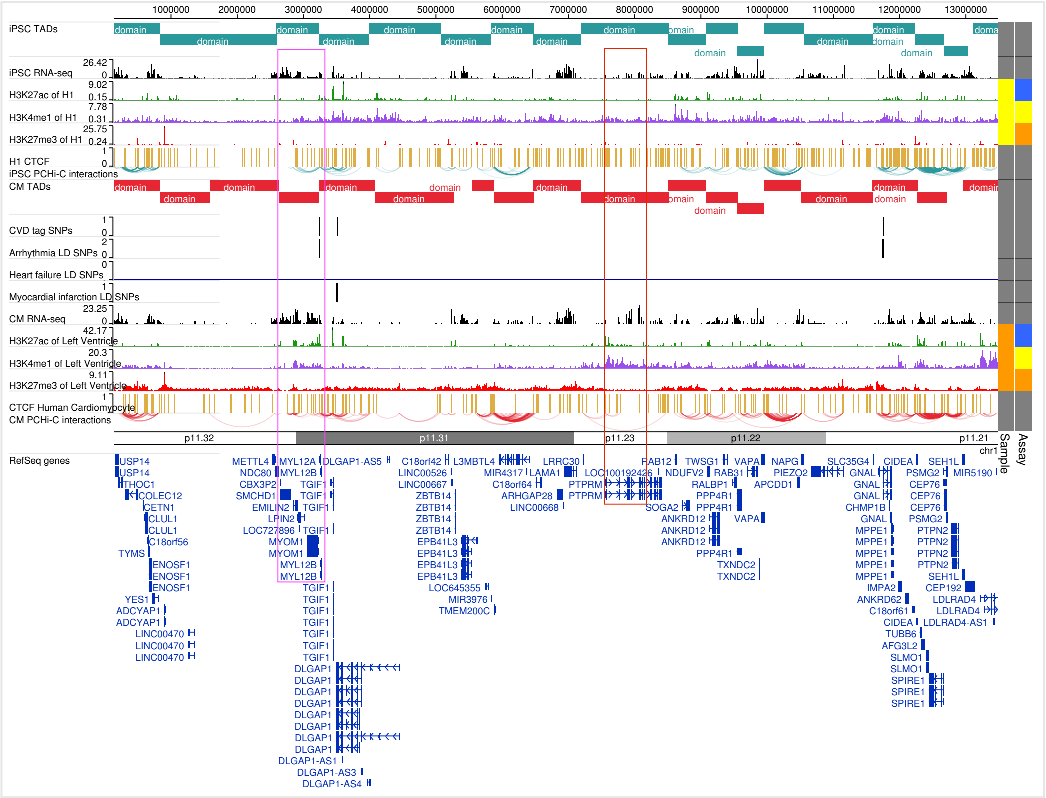

Supplementary Figure 3. Epigenomic landscape of candidate genes in the 18p11.32p11.22 *de novo* deletion locus. Plot of gene expression and epigenomic profiles (H3K27ac, H3K4me1 and H3K27me3) of induced pluripotent stem cells (H1 for iPSC, top) and iPSC-derived cardiomyocytes (left ventricle for CM, bottom) for genes encompassed in the 18p11.32p11.22 *de novo* deletion region from the Epigenome Roadmap Project/ENCODE using the WashU Epigenome Browser (v46.2).

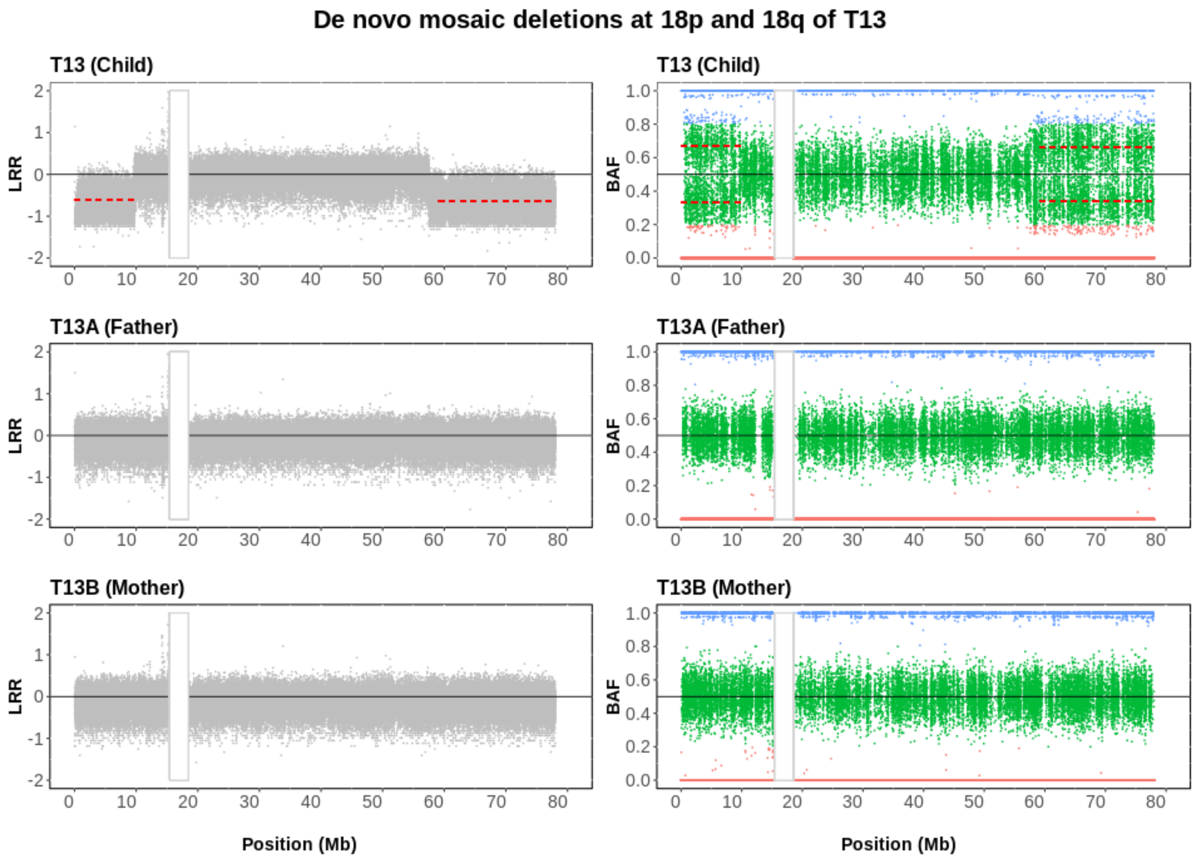

Supplementary Figure 4. Plot of log R ratio (LRR) and B allele frequency (BAF) of the *de novo* mosaic deletions at 18p and 18q of T13 trio. The mosaic deletions at 18p11.32p11.22 and 18q21.33q22.3 in the TOF proband (T13) are indicated by the small decrease in LRR from 0 to around -0.6 along with the narrow split in the middle BAF bands (top panel). A non-mosaic heterozygous deletion would have a larger decrease from 0 to -1 in LRR and no intermediate BAF bands. LRR and BAF bands are normal for both father (T13A; middle panel) and mother (T13B; bottom panel), indicating the absence of both deletions. Each point represents a SNP. In BAF plots the points are color-coded by genotype (red=AA, green=AB, blue=BB). The horizontal dashed red line is the mean value of LRR or BAF of the mosaic deletion region. The vertical gray rectangle represents the centromeric region.

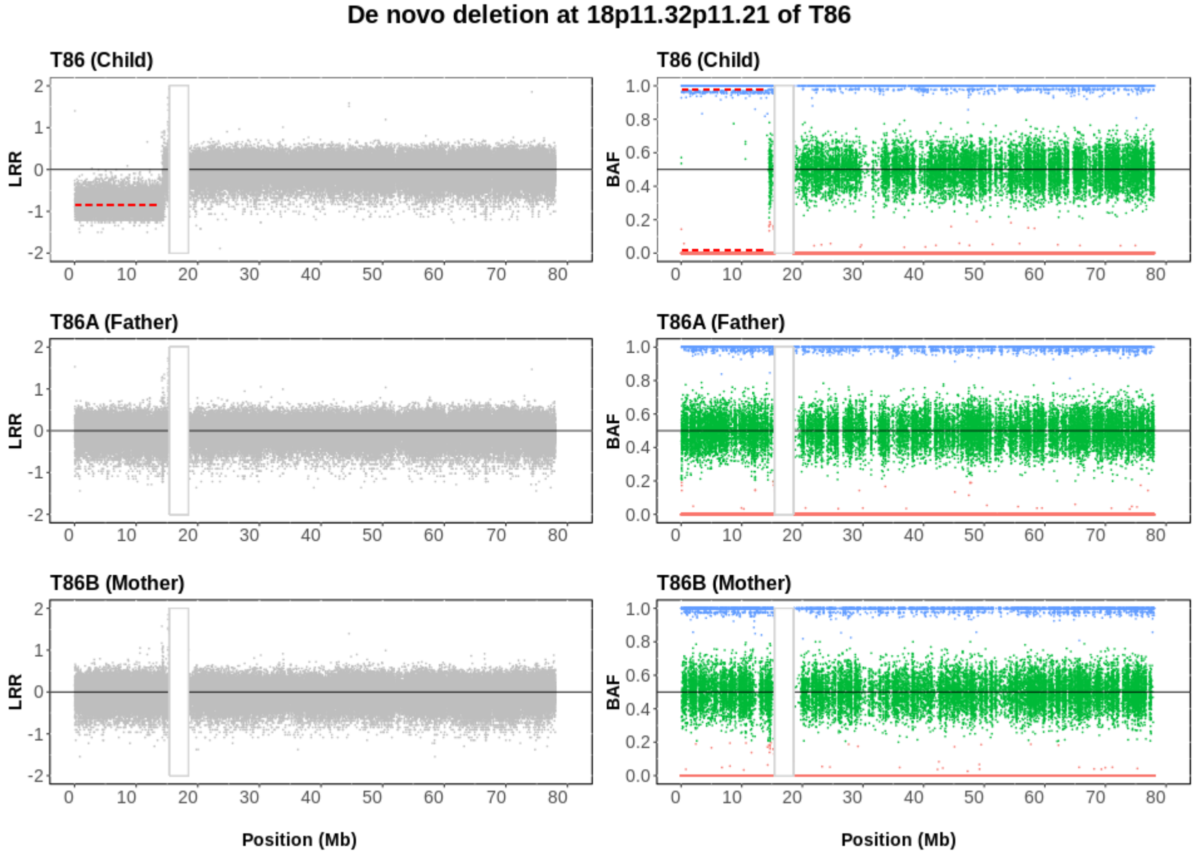

Supplementary Figure 5. Plot of log R ratio (LRR) and B allele frequency (BAF) of the *de novo* deletion at 18p11.32p11.21 of T86 trio. The deletion in the TOF proband (T86) is indicated by the decreased LRR from 0 to around -1 along with two BAF bands at 0 and 1. The LRR and BAF band are normal in both father (T86A; middle panel) and mother (T86B; bottom panel). Each point represents a SNP. In BAF plots the points are color-coded by genotype (red=AA, green=AB, blue=BB). The horizontal dashed red line is the mean value of LRR or BAF of the deletion region. The vertical gray rectangle represents the centromeric region.

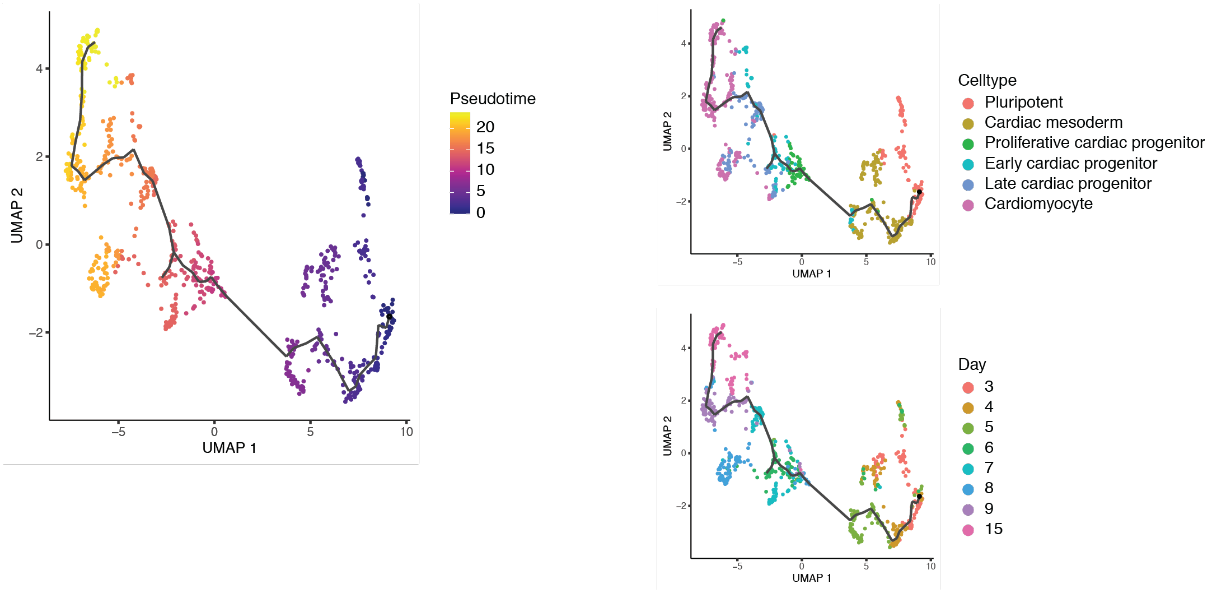

Supplementary Figure 6. Monocle 3 pseudotime trajectory based on UMAP dimensionality reduction. Single-cell trajectory of the hESC-cardiac differentiation constructed with Monocle 3. The uniform manifold approximation and projection (UMAP) dimensionality reduction plot is fitted with a principal graph, ordering the cells from early pseudotime to late pseudotime. Colors represent pseudotime values, cell type and time point of cell collection of the hESC cardiac differentiation protocol.
